# Round the Hospitals

**Published:** 1918-11-30

**Authors:** 


### ROUND THE HOSPITALS.

At the Investiture held at Buckingham Palace on November 23 the King conferred the decoration of the Royal Red Cross, Second Class, on Sister Jessie Ferguson, South African Military Nursing Service. The King has approved the award of the Military Medal to Miss Rosa Brain, staff nurse, T.F.N.S., for exceptional courage and devotion to duty during an hostile air-raid. One of the bombs wrecked the hut in which she was on duty, and with the greatest coolness she attended to all the patients in the ward though she herself was wounded.

---

Among the scenes of horror unveiled in the Austrian retreat none is more haunting to the imagination than the deserted hospital discovered by our troops "in a certain town." Every bed had its wounded occupant. In some the dead were lying. But the entire staff had fled. To the lasting shame of the Austrian doctors, not one was found to stay behind and minister to the helpless sufferers. And what of the nurses?

---

On Pound Day, November 19, at the London Homœopathic Hospital, the Countess of Donoughmore and Lady Perks opened the proceedings in the unavoidable absence of Lady Beatty in Scotland. There was a strong naval element owing to the presence of many sailor patients, whose dexterity of hand was shown in the pretty exhibition of articles made during their stay in the hospital. Gifts in kind of groceries and household necessaries amounted to 1,000 lb. in weight, and donations in

**ROUND THE HOSPITALS—*(continued)*.**

money to £125, besides £5 and more from a well-managed tea. Pound Days are so useful as an aid to housekeeping and so valuable in stimulating interest in hospital work that it is surprising they are not universal.

The Bolton stall topped the score in the great East Lancashire Bazaar. At one time it looked as if the Territorial stall was going to head the list, but by a spurt at the finish Bolton shot ahead and finally realised £1,050 out of the splendid total of £6,529 contributed to the Nation's Fund for Nurses. A marked feature in all these efforts is the opinion consistently expressed by members of the public that the nurse's lot requires to be alleviated. An uneasy conscience is stirring that too much has been required from, too little conferred on members of the profession, and the opportunity offered by the promoters of the Tribute Fund is being eagerly embraced.

Hampstead has gone, as usual, to the root of the matter in taking up the work of the National Council for Combating Venereal Diseases, and the annual report of the Hampstead Council of Social Welfare gives evidence of an advance along a seldom trodden road. A committee of women, under the chairmanship of Mrs. Perrin, were led, after consultation with inspectors, managers, and teachers, to the conclusion that some safeguard against the earliest physical tendencies toward impurity might be met by the appointment of school attendants to supervise the children of the infant departments whilst absent from the class-rooms. An offer was made to pay the salary and expenses of such an attendant, and place her at the disposal of the County Council for one year as an experiment. But the offer was declined.

The Council of Social Welfare are on strong ground when they insist that much more light needs to be thrown upon the hygienic conditions in our schools, and are insistent in their endeavours to stimulate public opinion in this direction. All who have intimate experience of the thousands of little children in our midst who may be described as "fallen," and whose redemption from sin and disease is slow, difficult, and costly, are agreed that in the vast majority of cases the mischief begins in the school lavatories. It is not merely in large town schools that this is the case, but in small villages. The evil is very wide-spread, and needs to be tackled with energy and perseverance. One or two very simple precautions would lessen, if not abolish, it.

Now is the time in institutions when a little forethought would obviate the heavy burdens which await the staff at Christmas. Everyone groans over the toll taken from scanty incomes by Christmas gifts to all and sundry, from the matron to the under-porter and ward scrubber. No one likes to hang back, but organisation can (and does in many places) relieve the situation. It is not too early for a notice to be put up deprecating gifts to the higher officials, and this should be rigidly enforced. For the manual workers a subscription list should be opened, as is done in clubs, and the proceeds divided. Everyone would be quit then for one contribution adapted to their means, and the voice of the horse-leech's daughter, too vociferous at all times but really unendurable towards Christmas, would be stilled.

Miss Clark, matron of the Whipps Cross Military Hospital, is to be congratulated on the completion of the fine new recreation hut for her patients which she has recently had the pleasure of opening with a suitably inscribed silver key. The ceremony devolved upon her by the express wish of the Guardians and of the Social Committee, who collected the funds as "a token of their appreciation of her devoted work." Forest House, where the wounded are accommodated, is in ideal surroundings among the glades of Epping Forest, and a large block of the adjoining West Ham Infirmary is also occupied by the wounded. The hut will prove beneficial to the convalescents in more ways than can be reckoned, and it may be permissible to express the hope that it can be retained as an adjunct to the infirmary long after the warriors are all recovered.

The Royal United Hospital, Bath, has been more than usually fortunate this year in the gifts bestowed by the Ladies' Working Association Guild. They were presented to the Mayor, as President of the hospital, and their value at present prices is estimated at between £400 and £500, considerably over double the usual amount. The Guild is in its twenty-fourth year, and numbers seventeen Vice-Presidents and 506 Associates, its President being Mrs. Handley. Miss Mason, the matron of the hospital, not only receives sufficient supplies of blankets, linen, and other necessaries for the hospital and the patients, but also for the Nurses' Home, the isolation ward, and the officers' quarters. It is good work, efficiently performed, which enlists, too, a wide measure of sympathy in the hospital's highest aims.

There is much searching of heart in respect of the impending closure of hospitals for the wounded as the pressure from overseas begins to decline. It is generally taken for granted that the authorities will proceed to close first the small auxiliary hospitals; but this is not the opinion of some at Headquarters, where it is believed that the auxiliary hospitals, which have acted as clearing-houses for the military establishments, will be needed to the end. Already steps are on foot to utilise for the benefit of the community in the approaching campaign against ill-health some of the buildings generously supplied for the use of the wounded. The impulse to give has been stimulated rather than exhausted by the efforts of the last four years, and there seems no reason to doubt a liberal continuance of voluntary aid in the nation's new tasks.

A new wing to accommodate a greatly enlarged nursing staff is to form part of the new Poor-Law Infirmary projected at Derby at an estimated cost of £100,000. The Local Government Board has sanctioned the scheme, which will be put in hand as

**ROUND THE HOSPITALS—(*continued*).**

soon as circumstances admit. It is very important that the Guardians in estimating the required accommodation for the nursing staff should have in view the certainty that nursing will be carried on in the near future on very different lines from the present. Not only will the size of the staff in relation to beds undergo a steady rise, but demands for more off-duty time, probably for one day off in seven, will increase in volume and will speedily be recognised on all sides as reasonable. New nursing homes should, in the interests of true economy, be planned with a view to subsequent enlargement, and should from the first take at least 20 per cent. more inmates than the existing number of nurses.

MISS WEARHAM, the nurse to whom the late Mrs. Barlow, of Bournemouth, left £1,500, has lost her case. It will be remembered that the will was disputed by Mrs. Barlow's relatives, who alleged that the legacy to the nurse and also one of £1,700 to the doctor were the result of undue influence. There was conclusive evidence that the old lady was kept away from her friends, under the exclusive influence of the nurse and doctor, who used it to their own advantage. The position of a nurse in attendance on the aged is a peculiarly delicate one. Although there are occasions when the patient rightly desires to reward one from whom all the comfort of the last declining years has flowed, yet the rules which govern their intercourse are perfectly clear, but to prevent all possibility of blundering they ought to be brought to the notice of every nurse as part of her training. To infringe them is to embark on one of the meanest paths open to a woman.

THE following letter from the Director-General Army Medical Services, addressed to the nursing staff of every military hospital, has been circulated to all Territorial matrons in Great Britain, France, and the East:—" War Office, 26th October, 1918. —The Director-General wishes it to be known in all the military hospitals how much he appreciates the unselfish devotion to their duty of the members of the Nursing Services at this time of emergency. He is aware that they are being very much overworked, and regrets that in spite of all the efforts which have been and are being made to procure more nurses, it is quite impossible, owing to the widespread epidemic of influenza, to send the necessary help which the needs of the hospitals demand. The admirable spirit of devotion to duty of all the ranks of the Nursing Services will be brought to the notice of the Nursing Board and the Army Council.—(Signed) E. WORTHINGTON, Colonel, A.D.G."

MANY of our readers doubtless had the opportunity of personally welcoming Princess Mary during her tour of hospital visits in France this week. Although officially her expedition is alluded to as an inspection, it is rather as a student that the Princess travels, and her previous experience as Commandant of her Voluntary Aid Detachment, as well as her work at Devonshire House and her duties in the wards at the Great Ormond Street Hospital, have all fitted her to make use of the opportunities extended to her during this crowded week. It is true of all such experiences that the spectator sees what she brings with her. The Princess, who has often accompanied her mother in visiting institutions for the sick, is already developing that trained eye which makes the Royal visits doubly valuable. She inherits the tradition that Royalty stands for the protection and uplifting of the sick, the maimed, and the poor, and her ideals of service and true womanliness can hardly fail to be widened by the study of woman's part behind the lines in the great war. She is having a splendid reception everywhere, and giving widespread pleasure by her spontaneous interest in every phase of work.

THE nursing profession was well represented at the Thanksgiving Service in St. Giles' Cathedral, Edinburgh, on the occasion of the visit of Their Majesties to Scotland on November 21. The Territorial Force Nursing Service was represented by Miss Gill, R.R.C., principal matron; Miss Millar, R.R.C., matron; and four of the sisters of the 2nd Scottish General Hospital, who were accorded a prominent place not far from the Royal pew. There were also present members of the Royal Naval Nursing Service, six sisters from the Royal Infirmary, and various other nurses, some of whom had come with wounded soldiers. In their fifteen-mile progress through the streets Their Majesties passed most of the principal hospitals—all decorated and beflagged in the brilliant sunshine. The nursing staff at Chalmers' Hospital had gathered a maximum number of sick at the windows to greet the Royal guests. The balconies of the Royal Infirmary were thronged with patients and nurses. The King and Queen responded heartily to their welcome, and looked with evident interest and pleasure on the building they knew so well.

LATER on, opposite the Longmore Hospital, the incurables and their nurses saw the blinded soldiers from the Scottish Blinded Soldiers' Hospital marshalled before Their Majesties' carriage, and heard the King say a few words of kindly encouragement to these poor men who could not see their Sovereign. After luncheon the *cortège* again proceeded through the crowded streets; the sun shone brightly, bells pealed out, flags waved, and even undemonstrative Scots shouted themselves hoarse with cheering. Here and there were members of the Scottish Women's First-Aid Corps with their stretchers, but the people were too full of joy over victory and their Sovereign's presence to think of fainting! Perhaps the prettiest hospital scene was at Flora Stevenson's School, now part of the 2nd Scottish General Hospital, where members of the T.F.N.S. and V.A.D.s in their picturesque uniforms were lined up in the open space round the building with the blue-suited wounded to cheer the King and Queen and the soldier Prince. All three beamed upon them, and seemed especially pleased at this demonstration of loyalty from the military hospital.

**ROUND THE HOSPITALS—*(continued)*.**

AFTER a painful illness lasting six weeks, Miss L. Atkinson, the popular matron of the Northampton General Hospital, passed away there on Friday, November 22. Miss Atkinson was trained under Miss Cummins at the Cumberland Infirmary, and on its completion she became assistant and then acting-matron there. She then went to the Liverpool Royal Infirmary as senior home sister, and later was appointed to the important post of assistant matron. She won golden opinions at both the Cumberland Infirmary and at the Liverpool Royal Infirmary, and Miss Cummins, under whom she had worked at Carlisle and at Liverpool, testified to her administrative and nursing talents. Miss Atkinson went to Northampton early in 1916, where her high character and standards soon won for her many friends, and the managers at once recognised her organising abilities, which have proved invaluable to the quality of the work done since she took office. She endeared herself to the nursing and domestic staffs, and was instrumental in the enforcement of many reforms which made for an all-round efficiency, and added much to the comfort of the establishment. She had the rare gift of making friends, and her death has caused sincere mourning at Liverpool, at Carlisle, and at the General Hospital, Northampton, where she was devotedly nursed during her last illness, as testified to by her sister. (See page 188.) A special memorial service, full of tender feeling and affectionate remembrance, was held in the chapel of the Royal Infirmary, Liverpool, on Sunday, when the Rev. J. Russell-Darbyshire, giving a farewell address, dwelt with grateful recognition on Miss Atkinson's unswerving loyalty, devotion to duty, forgetfulness of self, and singleness of purpose, which won all hearts. Her progress was remarkable, and her sudden and painful death, at an age when she had in the ordinary course many years of valuable service to the nursing profession before her, has cast a shadow wherever her work was known. Her life and work should inspire many of her late and former colleagues with the will to go and do likewise.

THE report of the sub-committee appointed on July 5, 1918, to examine into the conditions under which the nursing is carried on at the General Infirmary, Leeds, has just been issued. The recommendations of the Lady Superintendent are published in full, together with written statements furnished by a deputation (*a*) of sisters, (*b*) of nurses in training. Nearly all the matron's recommendations were accepted, and the estimated annual cost to the infirmary is £5,285. The sweeping changes inaugurated involve an increase of salary amounting to £495 a year to the present staff. An addition of forty members to the nursing staff was recommended, at an annual cost for salary of £820, and of board and lodging (estimated at £80 per head) of £3,200. Five extra maids are to be provided. The off-duty hours of sisters and nurses are largely increased, and the annual holiday lengthened to four weeks and three weeks respectively, with twelve off-days in addition. A new dining-room is to be secured for the staff, the old one being so completely inadequate as to form a fertile and just source of complaint. Many minor causes of friction are to be removed. The sisters are to have late dinner and more personal freedom, and night nurses are to have better meals, night duty occupying the last three months of the second year and the first six months of the third, with the annual holiday in between, instead of lasting as heretofore for twelve months.

THE spirit in which the committee, which consisted of eleven gentlemen, have conducted their inquiry is manifestly excellent. The policy of openly inviting suggestions from different ranks among the staff is most commendable, and the written statements of both sisters and nurses, which are well put together and moderate in tone, justified the innovation. It is evident that the work at the Leeds General Infirmary has been carried on lately under a grave condition of understaffing, and that the personal comfort of the nurses has been ignored in some important directions. The report shows that the authorities are entirely of one mind in their desire to put matters on a good basis, and there is every reason to hope the infirmary will take rank from now on as a really well-managed modern training-school. It is a pity that a new Nurses' Home cannot be secured at once. We hope that will come before very long.

INFLUENZA is still claiming its victims, and two members of the nursing staff of St. Thomas's Hospital have died from its effects. Miss Diana Marion Edgell, who died on November 22, is the second member of the nursing staff of St. Thomas's Hospital to succumb to pneumonia following influenza. The service was held first in the hospital chapel and afterwards in Brompton Cemetery. Miss Edgell was one of the three successful competitors for the sister-tutor scholarships given by the College of Nursing, whose success we announced in our issue of September 21. She had already taken up her course of training in Household and Social Science at King's College for Women. Her record at her training-school is that "her enthusiastic devotion to her work, her fine loyalty, her wholesome influence, her happy yet practical outlook on life, her earnest endeavour to apply herself to all that is best with a cheerful and unswerving steadfastness, won her the affection and esteem of her training-school. She was essentially fitted by character and education for the work ahead of her, and she brought to it ideals and a vision that raised good hopes in those who looked forward to the difficult days of reconstruction, and who sensed in her the ardour of a cause." Miss Edgell was buried on the 25th, the service being held first in the hospital chapel and afterwards in Brompton Cemetery. Miss Ellen Wace, one of five Nightingale nurses who qualified for the 1918 gold medal, has also died from pneumonia following influenza. She had successfully passed her final examinations, but had not yet completed her period of training to obtain the Nightingale certificate. She was held to be a most promising nurse, filled with a true sense of duty and a quiet devotion for her work, popular with her colleagues, trusted by those in authority, and worthily upholding the traditions of her training-school. Miss Wace was the niece of Dr. Wace, Dean of Canterbury.

